# Abnormal Elevation of the Expression of Costimulatory Molecule CD226 in Graves' Disease

**DOI:** 10.2174/0118715303337480250107052116

**Published:** 2025-02-12

**Authors:** Zhaowei Huang, Xuerong Liu, Tiantian Cai, Yanfei Jiang, Yuqing Wu, Xinwei Zhang, Rong-hua Song, Jin an Zhang

**Affiliations:** 1 Graduate School, Shanghai University of Traditional Chinese Medicine, Shanghai 201203, China;; 2 Department of Endocrinology, Shanghai University of Medicine & Health Sciences Affiliated Zhoupu Hospital, Shanghai 201318, China;; 3 Affiliated Hospital of Yan'an University, Yan'an 716000, China

**Keywords:** Graves’ disease, CD226, co-stimulatory molecule, PBMCs, CD4+, autoimmune thyroid disease, thyroid tissue

## Abstract

**Objective:**

This study aimed to explore the differential expression of the co-stimulatory molecule CD226 in lymphocytes from patients with New-Onset Graves' Disease (NOGD) and its correlation with clinical indicators.

**Methods:**

Sixty-eight participants were recruited for the discovery experiment (NOGD: healthy control (HC) = 39:29). Peripheral Blood Mononuclear Cells (PBMCs) were isolated. Flow cytometry was performed to detect CD226 expression on multiple lymphocyte subtypes. CD226 mRNA expression in PBMCs was detected by qPCR. Fifty-eight participants were recruited for the validation experiment (NOGD: HC=35:23). CD4^+^ T cells were isolated, and the level of CD226 mRNA in CD4^+^ T cells was detected. Five cases of each of Graves' disease (GD) thyroid and control thyroid were collected for CD226 immunohistochemical staining.

**Results:**

CD226 expression was the highest in monocytes (NOGD: 94.1% vs. HC: 94.8%) and the lowest in CD8^+^ T cells (NOGD: 65.3% vs. HC: 64.9%). Compared with HC, CD226 expression on the CD4^+^ T cells increased in the peripheral blood of NOGD patients and correlated with TPO-Ab. Meanwhile, CD226 mRNA levels were elevated in CD4^+^ T cells and positively correlated with TR-Ab. CD226 expression was significantly increased in the thyroid tissues of GD patients.

**Conclusion:**

This study demonstrates for the first time the elevated expression of CD226 in CD4^+^ T cells and thyroid tissue of NOGD. The abnormal elevation of CD226 is correlated with clinical indicators. It suggests that the co-stimulatory molecule CD226 is involved in the pathogenesis of GD.

## 1 INTRODUCTION

Graves’ Disease (GD), also known as toxic diffuse goiter, is a common thyroid-specific autoimmune disorder. It is characterized by hyperthyroidism and the production of autoantibodies against the thyroid-stimulating hormone receptor (TR-Ab). Some patients also develop eye lesions. The pathology of the disease is characterized by a massive infiltration of lymphocytes in the thyroid tissue, which produce cytokines, such as IFN-γ and TNF-α, to perpetuate the autoimmune inflammatory response [1, 2]. The overall prevalence of hyperthyroidism in the general population is about 1.3 percent, of which more than 80 percent is caused by GD. GD can occur at any age, but it is most common in women between the ages of 30 and 50, with a male-to-female ratio of about 1:4 [3-5]. The mechanism of the disease is not fully understood, and its treatment is limited to symptomatic therapy or surgery. Therefore, it is crucial to identify the factors that undermine immune tolerance in GD. Etiological treatments developed to address this factor will significantly contribute to improving the symptoms and quality of life of GD patients.

CD226 is a recently discovered immune co-stimulatory molecule belonging to the immunoglobulin superfamily. It is widely expressed on the surface of T cells, natural killer (NK) cells, monocytes, macrophages, and other cells. CD226 regulates the homeostasis of the immune system by competing for the ligands CD155 and CD112 with TIGIT, which transmits inhibitory signals. TIGIT is an immune checkpoint that suppresses both intrinsic and adaptive immunity through a variety of mechanisms. The balance of TIGIT/CD226 expression regulates the effector functions of T cells and NK cells [6, 7]. The intracellular domains of CD226 contain multiple serine and tyrosine phosphorylation sites that mediate activation signals to activate the immune system. CD226 molecule regulates T cell differentiation, proliferation, and cytokine secretion and enhances the ability of CD8^+^ T cells and NK cells to kill infected cells. In CD8^+^ T cells, CD226 promotes the formation of immune synapses by interacting with the CD155 expressed by Antigen-Presenting Cells (APC) [8, 9]. It was found that in TCR-activated CD4^+^ T cells, CD226 knockdown reduced T-bet expression and IFN-γ production [10, 11]. CD226 strongly promoted Th1 differentiation and IL-17 production. As an adhesion molecule, CD226 is involved in the transendothelial migration of effector memory cells, allowing the cells to leave the circulation and enter sites of inflammation. These cells or cytokines are closely related to the pathogenesis of GD. Rs763361 (G307S), a single nucleotide polymorphism locus of CD226, is the common cause of Type 1 Diabetes (T1D), Systemic Lupus Erythematosus (SLE), Rheumatoid Arthritis (RA), Systemic Sclerosis (SS), Multiple Sclerosis (MS), and other autoimmune diseases [12-15]. It is clear that CD226 promotes the development of many autoimmune diseases, but the role of this co-stimulatory molecule in GD is unclear. Therefore, we studied the differential expression of CD226 in New-Onset GD (NOGD) patients and its correlation with clinical indicators.

## 2 MATERIALS AND METHODS

### 2.1 Participants

The diagnosis of GD should be made by combining clinical manifestations and laboratory findings [16]. Clinical symptoms and signs include irritability, agitation, heat intolerance, excessive sweating, weight loss, muscle weakness, hyperphagia, and diffuse goiter. Laboratory tests include serum total thyroxine (TT4), free thyroxine (FT4), serum total triiodothyronine (TT3), free triiodothyronine (FT3), thyroid-stimulating hormone (TSH), TR-Ab, and thyroid peroxidase antibodies (TPO-Ab). All laboratory tests were performed in the central laboratory of Zhoupu Hospital. Participant exclusion criteria included age <18 years, history of antithyroid medication, history of thyroid hormone analog medication, history of thyroid radioisotope therapy, concomitant cancers, serious infections, serious cardiorespiratory disease (such as coronary artery disease, chronic obstructive pulmonary disease), and breastfeeding or pregnancy.

We recruited 39 NOGD patients and 29 age- and sex-matched healthy controls (HC) for the discovery experiments from June, 2020 to September, 2020. CD226 protein and CD226 mRNA expressions were detected in PBMCs. Another 35 NOGD patients and 23 healthy controls were used for the validation experiment from May, 2023 to September, 2023 to detect CD226 mRNA expression in CD4^+^ T cells. Validation experiments were used to further explore and validate the results of the discovery experiments and to avoid possible selection bias among the participants of the discovery experiments. The GD thyroid glands were obtained from five thyroidectomized GD patients, and the control thyroid tissues were obtained from the paracancerous tissues of five thyroid tumor patients. All patients were from the Department of Endocrinology, Shanghai University of Medicine and Health Sciences Affiliated Zhoupu Hospital. All healthy controls (normal thyroid ultrasound) were from the medical examination center of the same hospital. The study was approved by the Ethics Committee of Zhoupu Hospital (2023-NSFC-17-61030219504170039), and all participants signed an informed consent form.

### 2.2 PBMCs and CD4^+^ T Cell Isolation

Approximately 10 mL of venous blood was collected from each participant using heparin anticoagulant tubes. Peripheral Blood Mononuclear Cells (PBMCs) were isolated by Ficoll density gradient centrifugation according to the instructions of lymphocyte separation solution (DAKEWE, Beijing, China). Fresh blood was diluted with PBS in equal proportions, and then the diluted blood was slowly added to the lymphocyte isolation solution and centrifuged at 1500 rpm for 20 min. After centrifugation, the liquid surface was divided into three layers, and the PBMCs in the middle white membrane layer were aspirated in new centrifugal tubes, which were washed twice with PBS. After centrifugation at 1200 rpm for 5 minutes, the cells were resuspended with cell cryopreservation solution or PBS. The isolated PBMCs were stored at -80°C for flow cytometry experiments or immediately for further isolation of CD4^+^ T cells.

CD4^+^ T cells were isolated by magnetic bead separation and according to the instructions for CD4 MicroBeads reagent (Miltenyi Biotec Inc, Bergisch Gladbach, Germany). PBS resuspended PBMCs were subjected to cell counting; 10^8^ PBMCs were kept in 800 µL of buffer, then 200 µL of MACS CD14 MicroBeads were added and incubated at 4°C for 15 min. Cells were washed, and CD14^+^ monocytes were removed with an LD column. The LD column was placed in the magnetic field of the MACS separator, then the magnetic bead-labeled cells were placed on top of the LD column, and the unlabeled cells that passed through the magnetic field were collected. After centrifugation at 300 g for 10 min, the cells were resuspended in 160 µL of buffer, and 40 µL of MACS CD4 MicroBeads were added to incubate at 4°C for 15 min. The cells were washed, and a positive selection of CD4^+^ cells was performed using an LS column. The cell suspension was placed on top of the LS column to allow negative cells to pass through the magnetic field. The LS column was removed from the separator and placed on a new collection tube, 5 mL of buffer was added, and the positive cells were rinsed out using a plunger.

### 2.3 Flow Cytometry

The PBMCs samples were removed from the freezer at -80°C and quickly thawed in a water bath at 37°C to rejuvenate the cells. The recovered PBMCs were washed sequentially with 3 mL of 1640 medium and PBS and then resuspended in 15 μL of staining buffer. The cells were then labeled by adding 2 μL of different fluorochrome-conjugated antibodies, respectively, and incubated for 20 min in the greenhouse, protected from light. Antibodies added included CD14-BV510, CD16-eFluor450, CD56-eFluor450, CD3-BUV395, CD4-FITC, CD8-PE-Cy7, and CD226-APC (CD14-BV510 was produced from BioLegend, CD3-BUV395 was produced from BD, and other antibodies were obtained from Invitrogen). Finally, the cells were washed with pre-cooled washing buffer and resuspended with 50 μL of staining buffer and then detected by flow cytometry (BD Fortessa X20). Flow cytometry experimental data were analyzed with FlowJo software (version 10.6).

### 2.4 Real-Time Quantitative PCR

Total RNA from CD4^+^ T cells was extracted with TRIzol reagent (TaKaRa, Kusatsu, Shiga, Japan). RNA concentration and purity were determined, and cDNA was synthesized using a reverse transcription kit (TaKaRa, Kusatsu, Shiga, Japan). Finally, CD226 mRNA was determined on an ABI PRISM 7500 (Thermo Fisher, Waltham, MA, USA) according to the instructions of the qPCR kit TB Green Premix Ex Taq™ II (TaKaRa, Kusatsu, Shiga, Japan). The forward primer was GTGGAGTGGTTCAAGATCGGG, and the reverse primer was GCTTCCTTATGACCATGCCAT.

### 2.5 Thyroid Tissue Staining

**CD226 Immunohistochemical Staining (IHC):** Sections were treated with 1 × citrate buffer for antigen recovery and blocked for endogenous peroxidase. Sections were immunostained with primary antibody (DNAM-1 Polyclonal Antibody, Human, Immunoway, Plano, TX, USA, YT1381, 1:200) at 4°C overnight. On the next day, the samples were stained with a secondary antibody conjugated by horseradish peroxidase (Jackson ImmunoResearch Laboratories, West Grove, PA, USA, 1:10,000) and developed using a DAB peroxidase substrate kit. Neutral gum was used to seal the sections and then images were captured with a Leica microscope. Mean optical density (staining depth/area) was measured using ImageJ software to determine CD226 expression.

**Hematoxylin-Eosin Staining (HE):** Sections were sequentially stained with hematoxylin for 5 min, ethanol 1% hydrochloric acid for differentiation, and 0.6% ammonia to give a blue color to the nuclei, with intermediate rinsing with running water. Eosin staining was performed for 2 min to give a pink color to the cytoplasm, followed by dehydration and transparency with different concentrations of ethanol and xylene.

### 2.6 Statistical Analysis

Statistical analyses were performed using *SPSS25.0*. Continuous variables with a normal distribution and homogeneity of variance were represented by (*X ± S*), and comparisons between groups were performed by *t*-test. Continuous variables that do not conform to the normal distribution are represented by the median (interquartile range) (M (P25-P75)), and comparisons between groups were made by the Mann-Whitney U test. Categorical variables are expressed in terms of quantity and percentage (n, %), and the χ^2^ test was used. In the statistical tests of the above methods, *P* < 0.05 was considered significant.

## 3 RESULTS

### 3.1 Elevated Expression of CD226 in CD4^+^ T Cells

**Fig. (1):**
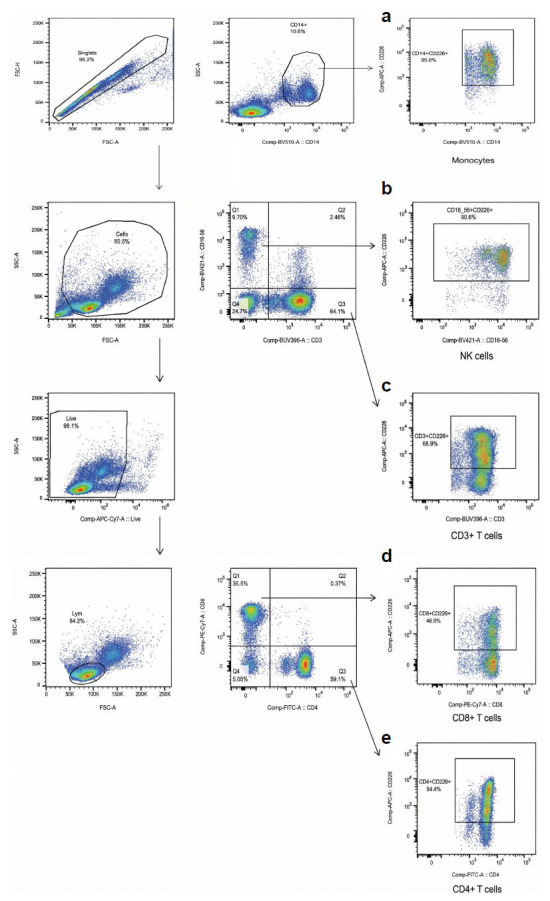
Gating strategy for flow cytometry analysis. (**a**) Monocytes (CD14^+^). (**b**) NK cells (CD3^-^CD16^+^CD56^+^). (**c**) CD3^+^ T cells (CD3^+^CD16^-^CD56^-^). (**d**) CD8^+^ T cells (CD3^+^CD4^-^CD8^+^). (**e**) CD4^+^ T cells (CD3^+^CD4^+^CD8^-^).

**Fig. (2):**
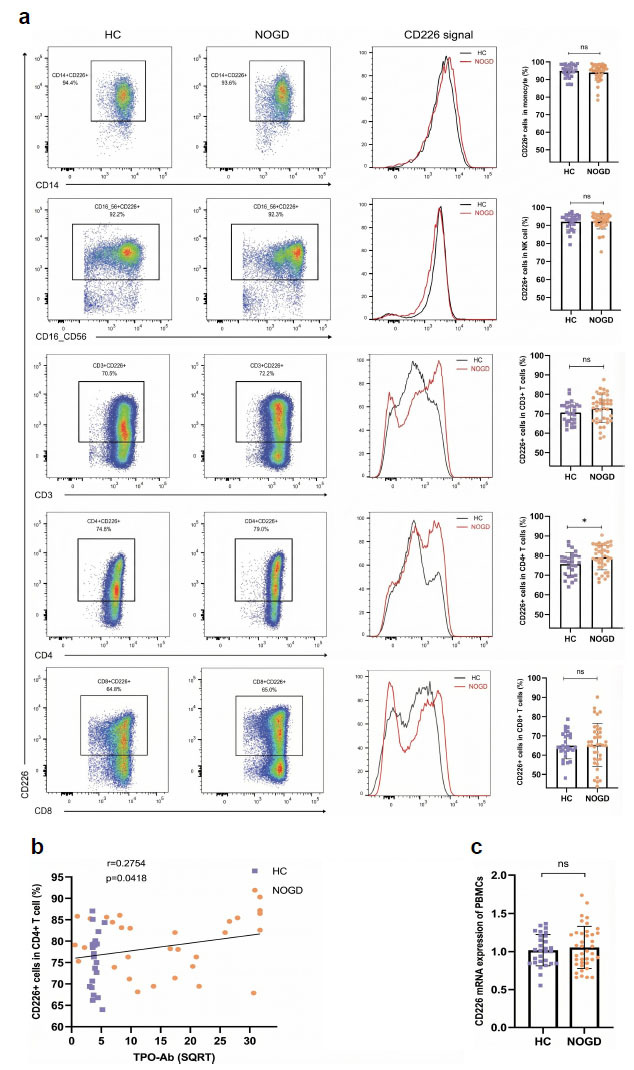
Elevated expression of CD226 in CD4^+^ T cells. (**a**) Expression of CD226 in different lymphocyte subpopulations. (**b**) CD4^+^ T cell CD226 expression correlated with TPO-Ab levels. (**c**) CD226 mRNA expression in PBMCs. (ns, *P* ≥ 0.05; *, *P* < 0.05; HC: Healthy Control, NOGD: New-Onset Graves' Disease, SQRT: Square Root, PBMCs: Peripheral Blood Mononuclear Cells).

A total of 68 participants were included in the discovery experiment (Table **1**). Multicolor flow cytometry was performed to detect CD226 expression in various lymphocyte subtypes (Fig. **1**). The results showed that CD226 expression was the highest in monocytes (NOGD:94.1% vs. HC:94.8%) and the lowest in CD8^+^ T cells (NOGD:65.3% vs. HC:64.9%). The difference in CD226^+^ cells in monocytes, NK cells, CD3^+^ T cells, and CD8^+^ T cells was not statistically significant in NOGD compared to HC. There was a significant increase in CD226^+^ cells and CD4^+^ T cells in the NOGD compared to the HC (Fig. **2a**). There was a positive correlation between CD4^+^CD226^+^ cells and TPO-Ab levels (r=0.2754) (Fig. **2b**). No differences in CD226 mRNA were found in PBMCs (Fig. **2c**). Among the various lymphocyte subtypes, only CD4^+^ T cells showed significant differences in CD226 expression. Therefore, further studies were conducted by isolating CD4^+^ T lymphocytes in the validation experiment.

### 3.2 Elevated Expression of CD226 mRNA in CD4^+^ T Cells

**Fig. (3):**
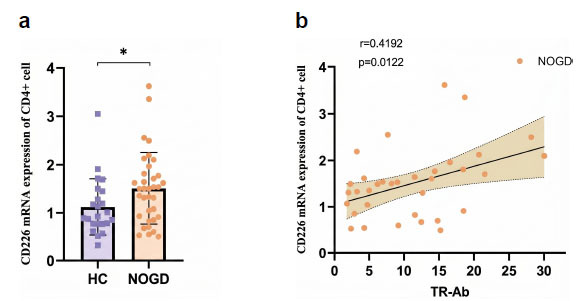
Elevated expression of CD226 mRNA in CD4^+^ T cells. (**a**) CD226 mRNA expression in CD4^+^ T cells. (**b**) CD226 mRNA expression in CD4^+^ T cells was positively correlated with TR-Ab levels. (*, *P* < 0.05; HC: Healthy Control, NOGD: New-Onset Graves' Disease, TR-Ab: Thyroid Stimulating Hormone Receptor Antibodies).

A total of 58 individuals participated in the validation experiments (Table **2**). qPCR results of CD4^+^ T cells showed that CD226 mRNA expression was significantly higher in NOGD compared to HC (Fig. **3a**). Moreover, CD226 mRNA expression was positively correlated with TR-Ab levels in NOGD patients (r = 0.4192) (Fig. **3b**).

### 3.3 Elevated Expression of CD226 in Thyroid Tissue

**Fig. (4):**
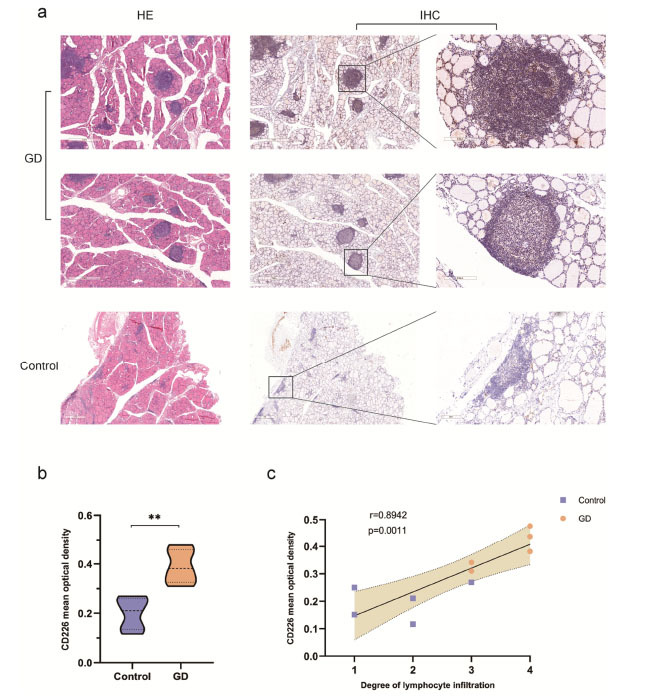
Elevated expression of CD226 in thyroid tissue. (**a**) Representative images of thyroid tissue staining. (**b**) CD226 staining intensity increased. (**c**) CD226 mean optical density was positively correlated with the degree of lymphocytic infiltration. (**, *P* < 0.01; HE: Hematoxylin-Eosin Staining, IHC: Immunohistochemical Staining; GD: Graves’ Disease).

HE results showed that there was more lymphocyte infiltration in the GD thyroid tissue than in the control, and the normal follicular structures were replaced by germinal centers formed by plasma cells and lymphocytes (Fig. **4a**). The IHC results showed that the CD226 staining intensity was higher in GD than in control, especially in the germinal centers where lymphocytes congregated (Fig. **4a**). Brown staining indicated CD226 protein positivity. The CD226 mean optical density of GD was significantly higher than that in the control (Fig. **4b**). The CD226 mean optical density was positively correlated with the degree of lymphocyte infiltration (r = 0.8942) (Fig. **4c**).

## 4 DISCUSSION

Hyperthyroidism is caused by an increase in self-synthesis and secretion of thyroid hormones. It is a sign of overactive thyroid function. Currently, antithyroid drugs (ATDs), radioactive iodine (RAI), and surgical resection are symptomatic treatments for GD and are associated with additional risks [17-19]. ATDs can not only cause serious adverse reactions, such as granulocytosis and drug-induced liver injury, but the disease also relapses easily after withdrawal. RAI is more likely to lead to permanent hypothyroidism. Surgical removal carries a risk of hypoparathyroidism and recurrent laryngeal nerve damage, and patients require lifelong thyroid hormone replacement therapy. Therefore, the determination of the pathogenesis of GD is not only urgent but also fascinating, and the abnormal activation of immune cells has become a research hotspot. Previous studies have reported that CD4^+^ T cells, including Th1, Th2, Th17, Th22, and Treg, play an important role in the immune mechanism of GD [20-22]. In the present study, the expression of the immune co-stimulatory molecule CD226 was found to be increased in NOGD from three dimensions: affected thyroid tissue, peripheral lymphocytes, and genetic information mRNA. These CD226 abnormalities also correlated with clinical indicators. These results suggest that CD226 is involved in the pathogenesis of GD.

CD4^+^ T lymphocytes participate in the immune response by differentiating into different subpopulations and releasing different cytokines. Large amounts of autoimmune antibodies are present in the thyroid gland of patients with GD. CD4^+^ T cells are involved in B cell differentiation, helping B cells in lymphoid follicles to proliferate and differentiate into autoantibody-secreting plasma cells. This promotes the formation of germinal centers and the production of high-affinity antibodies, which are necessary for the development of chronic immune inflammation [23-27]. Studies have reported that Th1 cells are abnormally increased in autoimmune thyroid disease [28]. Serum levels of IL-12 and IL-18 are significantly higher in GD patients than in healthy controls [29]. These Th1 cytokines further induce naive CD4^+^ T cells to differentiate into Th1 cells [30]. Th1 cells play an important role in GD by activating macrophages and cytotoxic lymphocytes, which directly damage thyroid follicular cells. Th2 produces cytokines, such as IL-4, IL-5, IL-6, and IL-10, which can stimulate B cells to differentiate into mature plasma cells, and plasma cells produce a large amount of TR-Ab, resulting in hyperthyroidism [31-34]. Studies have found that during the occurrence of GD, thyroid cells themselves also produce IL-1, IL-6, TNF-α, TGF-β, and other cytokines, thus aggravating the inflammatory process [35-37]. The important role of Th17 cells and their active molecule IL-17 in the pathogenesis of many autoimmune diseases, including GD, has been demonstrated [38, 39]. IL-17 is an early initiator of the inflammatory response and can induce the secretion of many pro-inflammatory cytokines and chemokines. It has been found that patients with Autoimmune Thyroid Disease (AITD) have an increased number of Th17 cells in the circulating blood and thyroid gland [40]. A microenvironment that facilitates the differentiation of CD4^+^ T cells to Th17 may be prevalent in patients [41]. Patients with AITD have significantly increased levels of IL-6, IL-15, IL-23, and RORC2 in the peripheral blood [42, 43]. Activated CD4^+^ T lymphocytes also differentiate into Th22 cells. IL-22 is synergistically pro-inflammatory with IL-17, TNF-α, and IFN-γ [44]. In a word, the proliferation and differentiation of CD4^+^ T cells play a key role in the development of GD. However, the high expression of CD226 on CD4+T may be one of the reasons that help its abnormal activation.

CD226 is a member of the immunoglobulin superfamily and is localized on the surface of the cell membrane. Its extracellular domain binds the ligands CD155 and CD112, and its intracellular domain binds Lymphocyte Function-Associated Antigen-1 (LFA-1) and the junction protein Grb2 to transmit activation signals [45-47]. In recent years, it has been found that CD226-mediated activation signals are crucial for the activation and function of CD4^+^ T cells and NK cells [48, 49]. CD226 regulates various aspects of T cell function by binding to CD155. Moreover, CD226 binds to CD155 and promotes the phosphorylation of Forkhead Box O1 (FOXO1). FOXO1 plays an important role in NK cells, CD8^+^ T cells, and Treg cells and is a widely expressed transcription factor [50-53]. Phosphorylated FOXO1 is translocated to the cytoplasm for degradation. FOXO1 is a negative regulator of NK cell effector function, so CD226 binding to D155 promotes NK cell activity [54-58]. Similarly, CD226-mediated FOXO1 may also regulate T cell function. FOXO1 inhibits T-bet, a key transcription factor in CD4^+^ and CD8^+^ T cells that regulates multiple effector mechanisms [59-61]. In addition, Eomesodermin (EOMES) and Transcription Factor 7 (TCF7), key transcription factors involved in T cell memory, are also targets of FOXO1 repression [62-67]. FOXO1 regulates FOXP3 and is, therefore, important for Treg cell development and differentiation. Inactivation of FOXO1 results in reduced inhibitory activity of Treg cells [68-74]. In conclusion, overactivation of the immune co-stimulatory molecule CD226 not only activates NK cells and effector T cells but also suppresses Treg cell function. This leads to hyperfunction of the entire immune system.

However, the role of this immune molecule in GD remains unclear. In the present study, we found an increased expression of CD226 in the thyroid gland and peripheral blood CD4^+^ lymphocytes of GD. As a co-stimulatory molecule, CD226 enhances T cell activity and improves the proinflammatory ability of effector T cells in the inflammatory response. CD226 promotes Th1 differentiation and enhances the production of IFN-γ by naïve T cells [75, 76]. Expression of CD226 is increased in Th17-polarized naïve T cells. CD226 is indispensable for Th17 cell production and promotes IL-17 secretion [77, 78]. Studies on other autoimmune inflammatory diseases also support the pro-inflammatory activity of CD226. The expression of CD4^+^CD226^+^ cells is up-regulated in a mouse model of autoimmune encephalomyelitis (EAE), and blocking CD226 by anti^-^CD226 monoclonal antibodies can delay the onset of EAE. At the same time, mice with CD226 knocked out had reduced infiltration of Th17 cells in the central nervous system and increased Treg cells, thus reducing the occurrence of EAE [79]. In terms of pSS, CD4^+^CD226^+^ cells were found to be significantly higher in the PBMCs of patients than in normal controls and were closely correlated with the disease activity of patients [80, 81]. Notably, *in vitro* experiments from this study showed that the effector function of CD4^+^CD226^+^ T cells was higher than that of CD4^+^ CD226-T cells. Combined with our findings, we speculate that highly expressed CD226 molecules may promote the activation, proliferation, and differentiation of CD4^+^ T cells. Furthermore, the enhanced effector function of CD4^+^CD226^+^ T cells promotes GD development.

The limitations of this study are as follows: first, this study only elucidated the aberrant expression of CD226 in NOGD, and it would have been better if there had been a time-dimensional comparison of the changes in CD226 during the development of GD. Second, although an increase in CD4^+^CD226^+^ cells was found, the functional changes of such cells were not further explored. Certainly, this will be our next goal.

## CONCLUSION

In conclusion, our study revealed for the first time that the expression of the immune co-stimulatory molecule CD226 was increased in GD thyroid tissues and peripheral blood CD4^+^ T lymphocytes. Thus, CD226 may be involved in the pathogenesis of GD.

## Figures and Tables

**Table 1:** Clinical characteristics of the participants in the discovery experiment.

Variables	NOGD (n=39)	HC (n=29)	*P-*value
Age (years)	34.69 ± 9.10	33.45 ± 6.02	0.639
Female, n (%)	21 (53.8)	16 (55.2)	0.914
BMI (Kg·m^-2^)	22.36 ± 3.35	21.68 ± 3.37	0.815
Smoke, n (%)	9 (23.1)	6 (20.7)	0.814
TT3 (nmol·L^-1^)	6.03 ± 2.56	/	/
TT4 (nmol·L^-1^)	252.13 (197.12-303.01)	/	/
FT3 (pmol·L^-1^)	20.78 (13.16-30.72)	/	/
FT4 (pmol·L^-1^)	34.45 ± 12.48	/	/
TSH (uIU·mL^-1^)	0.002 (0.001-0.010)	/	/
TG (ng·mL^-1^)	1.60 (1.00-61.12)	/	/
TG-Ab (U·mL^-1^)	35.01 (8.02-183.58)	/	/
TR-Ab (IU·L^-1^)	9.50 (4.54-22.60)	0.37 (0.25-0.77)	<0.001*
TPO-Ab (U·mL^-1^)	271.02 (49.14-698.58)	13.82 (12.07-16.78)	<0.001*

**Note:** NOGD: New-Onset Graves' Disease; HC: Healthy Control; TT3: Total Triiodothyronine; TT4: Total Thyroxine; FT3: Free Triiodothyronine; FT4: Free Thyroxine; TSH: Thyroid Stimulating Hormone; TG: Thyroglobulin; TG-Ab: Thyroglobulin Antibodies; TR-Ab: Thyroid Stimulating Hormone Receptor Antibodies; TPO-Ab: Thyroid Peroxidase Antibodies. * Indicates a significant difference between the two groups.

**Table 2:** Clinical characteristics of participants in the validation experiment.

Variables	NOGD (n=35)	HC (n=23)	*P-*value
Age (years)	37.14 ± 12.55	34.57 ± 9.49	0.639
Female, n (%)	22 (53.8)	13 (56.5)	0.914
BMI (Kg·m^-2^)	21.53 ± 2.98	21.57 ± 2.88	0.815
Smoke, n (%)	8 (22.9)	4 (17.4)	0.814
TT3 (nmol·L^-1^)	6.30 ± 2.12	/	/
TT4 (nmol·L^-1^)	238.90 ± 65.59	/	/
FT3 (pmol·L^-1^)	25.41 ± 12.47	/	/
FT4 (pmol·L^-1^)	43.69 (33.80-68.20)	/	/
TSH (uIU·mL^-1^)	0.005 (0.001-0.005)	/	/
TG (ng·mL^-1^)	2.85 (1.00-46.43)	/	/
TG-Ab (U·mL^-1^)	67.95 (17.38-333.25)	/	/
TR-Ab (IU·L^-1^)	9.19 (4.29-15.90)	0.34 (0.25-0.66)	<0.001*
TPO-Ab (U·mL^-1^)	166.96 (12.59-572.25)	/	/

**Note:** NOGD: New-Onset Graves' Disease; HC: Healthy Control; TT3: Total Triiodothyronine; TT4: Total Thyroxine; FT3: Free Triiodothyronine; FT4: Free Thyroxine; TSH: Thyroid Stimulating Hormone; TG: Thyroglobulin; TG-Ab: Thyroglobulin Antibodies; TR-Ab: Thyroid Stimulating Hormone Receptor Antibodies; TPO-Ab: Thyroid Peroxidase Antibodies, * Indicates a significant difference between the two groups.

## Data Availability

The data that support the findings of this study are available from the corresponding author [JZ] upon reasonable request.

## LIST OF ABBREVIATIONS

NOGD: New-onset Graves' Disease
HC: Healthy Control
PBMCs: Peripheral Bblood Mononuclear cells
GD: Graves' Disease
T1D: Type 1 Diabetes
SLE: Systemic Lupus Erythematosus
RA: Rheumatoid Arthritis
SS: Systemic Sclerosis
MS: Multiple Sclerosis
TT4: Serum Total Thyroxine
FT4: Free Thyroxine
TT3: Serum Total Triiodothyronine
FT3: Free Triiodothyronine
TSH: Thyroid- Stimulating Hormone
TPO-Ab: Thyroid Peroxidase Antibodies
ATDs: Antithyroid Drugs
RAI: Radioactive Iodine
AITD: Autoimmune Thyroid Disease
FOXO1: Forkhead Box O1
EOMES: Eomesodermin
TCF7: Transcription factor 7

## STANDARDS OF REPORTING

Strobe guidelines were followed.
